# Left atrial spontaneous echo contrast occurring in patients with low CHADS_2_ or CHA_2_DS_2_-VASc scores

**DOI:** 10.1186/s12947-020-00213-2

**Published:** 2020-08-01

**Authors:** Kanako Akamatsu, Takahide Ito, Michishige Ozeki, Masatoshi Miyamura, Koichi Sohmiya, Masaaki Hoshiga

**Affiliations:** Department of Cardiology, Osaka Medical College, Takatsuki, Osaka, 569-8686 Japan

**Keywords:** Atrial fibrillation, Spontaneous echo contrast, Transesophageal echocardiography, CHADS_2_ score, CHA_2_DS_2_-VASc score

## Abstract

**Background:**

Left atrial spontaneous echo contrast (LASEC) is common in patients with atrial fibrillation (AF), although scarce information exists on LASEC occurring in nonvalvular AF patients who have low thromboembolic risk scores. We therefore examined prevalence and determinants of LASEC under low CHADS_2_ or CHA_2_DS_2_-VASc scores in these patients.

**Methods:**

Among 713 patients who underwent transesophageal echocardiography, 349 with a CHADS_2_ score < 2 (CHADS_2_ group) (93 women, mean age 65 years) and 221 with a CHA_2_DS_2_-VASc score < 2 (CHA_2_DS_2_-VASc group) (39 women, mean age 62 years) were separately examined for clinical and echocardiographic findings.

**Results:**

LASEC was found in 77 patients of CHADS_2_ group (22%) and in 41 of CHA_2_DS_2_-VASc group (19%). Multivariate logistic regression analysis, adjusted for several parameters including non-paroxysmal AF, LA enlargement (LA diameter ≥ 50 mm), left ventricular (LV) hypertrophy, and an elevated B-type natriuretic peptide (BNP) (BNP ≥200 pg/mL) revealed that for CHADS_2_ group, non-paroxysmal AF (Odds ratio 5.65, 95%CI 3.08–10.5, *P* < 0.001), BNP elevation (Odds ratio 3.42, 95%CI 1.29–9.06, *P* = 0.013), and LV hypertrophy (Odds ratio 2.26, 95%CI 1.19–4.28, P = 0.013) were significant independent determinants of LASEC, and that for CHA_2_DS_2_-VASc group, non-paroxysmal AF (Odds ratio 3.38, 95%CI 1.51–7.54, *P* = 0.003) and LV hypertrophy (Odds ratio 2.53, 95%CI 1.13–5.70, *P* = 0.025) were significant independent determinants of LASEC.

**Conclusions:**

LASEC was present in a considerable proportion of patients with nonvalvular AF under low thromboembolic risk scores. Information on AF chronicity, BNP, and LV hypertrophy might help identify patients at risk for thromboembolism, although large-scale studies are necessary to confirm our observations.

## Background

There are numerous reports that left atrial spontaneous echo contrast (LASEC) is one of the strongest predictors of intraatrial thrombosis and subsequent thromboembolism [1–4]. Thromboembolic (TE) risk scores typified by CHADS_2_ and CHA_2_DS_2_-VASc have been used for the past decade to assess TE risk and to guide prophylactic anticoagulation in patients with nonvalvular atrial fibrillation (AF) [5]. Studies on the association of transesophageal echocardiography (TEE) findings with CHADS_2_ or CHA_2_DS_2_-VASc scores in nonvalvular AF patients have shown a trend of which the greater score of CHADS_2_ or CHA_2_DS_2_-VASc, the more likely LASEC to be observed [6–8]; however, a certain number of patients are found to have LASEC despite low scores levels [6–10].

Generally, patients with non-paroxysmal AF who have chronic heart failure are in the predisposing condition to left atrial (LA) thrombus formation [11, 12], and particularly, those with increased LA size, left ventricular (LV) systolic dysfunction, and reduced LA appendage (LAA) velocity are most likely to be associated with LASEC and/or LA thrombus [13–16]. Scarce information, however, has existed on LASEC occurring in AF patients who have low TE risk scores [9, 10]. We therefore examined prevalence and determinants of the presence of LASEC in nonvalvular AF patients with low CHADS_2_ or CHA_2_DS_2_-VASc scores.

## Materials and methods

### Study population

We reviewed echocardiography reports, including digitized cine-loop images, and clinical charts on 713 patients with nonvalvular AF who underwent TEE between 2012 and 2018 in Osaka Medical College Hospital. TEE was performed in order to screen intracardiac thrombosis prior to pulmonary vein isolation procedure and/or direct cardioversion. There were 493 men and 220 women with a mean age of 67 years. Patients with rheumatic/degenerative mitral valve disease, congenital heart disease, and those in whom echocardiography and/or laboratory data considered to be important for the current analysis, particularly the B-type natriuretic peptide (BNP) and left ventricular (LV) ejection fraction, were lacking were excluded.

**Fig. 1:**
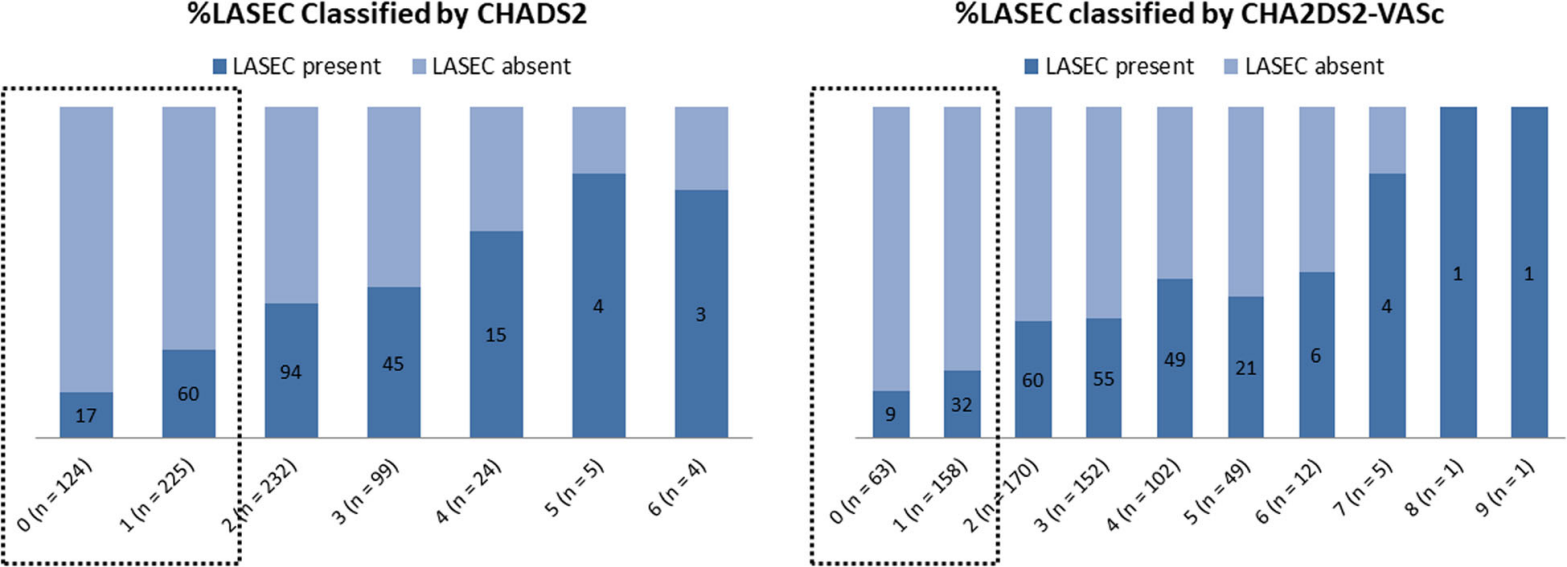
Percentages of the presence of LASEC classified by either CHADS_2_ (left) or CHA_2_DS_2_-VASc (right) score. Subgroups in the rectangles for each graph are those analyzed in the present study

Figure 1 shows percentages of the presence of LASEC classified by CHADS_2_ and CHA_2_DS_2_-VASc scores in the 713 patients. Overall, the incidence of LASEC was found to increase accordingly with increases in CHADS_2_ and CHA_2_DS_2_-VASc scores (*P* < 0.001 for both). In the present study, following results were all drawn separately for the 2 groups: 349 patients with a CHADS_2_ score < 2 (CHADS_2_ group); and 221 with a CHA_2_DS_2_-VASc score < 2 (CHA_2_DS_2_-VASc group).

This study was approved by the Ethics Committee of Osaka Medical College with notification for guaranteed withdrawal of participants on the website providing means of “opt-out” (No. 2194–01).

### Echocardiography

Ultrasound machines used were Vivid 7 Dimension and Vivid E9 with the phased array probes for both transthoracic echocardiography and TEE (GE-Vingmed, Horten, Norway). LA diameter, and LV dimensions and wall thickness were measured under 2-dimensional image guidance. LV ejection fraction was obtained with the modified Simpson’s rule in the 2- and 4-chamber views, and an ejection fraction < 50% was defined as LV systolic dysfunction. LV mass was calculated using the Devereux formula, indexed by the body surface area to draw LV mass index. LV mass index ≥115 g/m^2^ in men and ≥ 95 g/m^2^ in women were considered as the presence of LV hypertrophy [17]. The severity of mitral regurgitation was determined semi-quantitatively using color-flow mapping.

Standard multiplane TEE was performed using the same ultrasound machines with 6Tc and 6VT-D probes, respectively. The entire LA cavity was thoroughly examined for LASEC and LA thrombus with the gain setting being adjusted for optimal analysis. Attention was paid to differentiate the LAA thrombus from pectinate muscles [18]. TEE images, on a routine basis, were stored as cine-loops for the subsequent analysis. The severity of LASEC was categorized as being absent, mild or severe on the basis of the system described by Daniel et al. and Beppu et al. [19, 20]. Mild LASEC was defined as being present if dynamic echoes were seen only with high gain, whereas severe LASEC was present if spontaneous contrast was noted even with low gain.

To evaluate reproducibility of LASEC severity, 30 cases that were randomly selected from our population, including severe (*n* = 4), mild (*n* = 12), and none (*n* = 14), were analyzed by 2 independent experienced observers. The concordance rate (κ) for the corresponding LASEC severity was 0.93.

LAA velocity was also obtained with the pulsed Doppler sample volume 1 to 2 cm positioned inside the LAA orifice, averaged over 3 and 5 consecutive cardiac cycles in case of patients in sinus rhythm and of those in AF, respectively.

### Thromboembolic risk scores

CHADS_2_ score was calculated by giving 1 point each for congestive heart failure, hypertension, age ≥ 75 years, and diabetes, and 2 points for prior stroke or transient ischemic attack [21], and patients with a CHADS_2_ score < 2 were classified into the “low risk” category (CHADS_2_ group) [9, 22]. CHA_2_DS_2_-VASc score was calculated by giving 1 point each for congestive heart failure or LV systolic dysfunction (ejection fraction < 40%), hypertension, diabetes, vascular disease, age 65 to 74 years, and female gender, and 2 points for prior stroke or transient ischemic attack and for age ≥ 75 years [5], and patients with a CHA_2_DS_2_-VASc score < 2 were classified as “low risk” (CHA_2_DS_2_-VASc group) [9, 22].

Besides, we calculated HAS-BLED score (Hypertension, Abnormal Renal/Liver Function, Stroke, Bleeding History or Predisposition, Labile International Normalized Ratio [INR], Elderly, and Drugs/Alcohol) to assess the coagulation/bleeding status of the patients [23]. We gave 0 point of “Labile INR” to all patients who had been taking DOACs.

### Clinical definitions

Abnormalities of some clinical and echocardiographic parameters were determined as follows. Based on K/DOQI clinical practice guidelines [24], renal dysfunction was defined as an estimated glomerular filtration rate (eGFR) < 60 mL/min/1.73m^2^. BNP ≥200 pg/mL was considered clinically significant in accordance with the statement guideline by the Japanese Heart Failure Society (www.asas.or.jp/jhfs/english/outline/guidelines_20180822.html). LA enlargement and LAA dysfunction were defined as LA diameter ≥ 50 mm and LAA velocity < 20 cm/s, respectively [2, 25].

### Statistical analysis

Continuous variables were expressed as mean ± SD and categorical variables as percentages. Comparisons of categorical variables were performed using the chi-square test or Fisher’s exact test as appropriate. Univariate and multivariate logistic regression analyses were introduced to predict determinants of LASEC for both CHADS_2_ and CHA_2_DS_2_-VASc groups. All analyses were performed using JMP Pro ver. 14.0 (SAS Institute, Cary, NC). A *P* values < 0.05 was considered significant.

## Results

### Clinical and echocardiographic characteristics of the patient groups

Clinical characteristics of CHADS_2_ and CHA_2_DS_2_-VASc groups are presented in Table 1. With the exception of age and gender distribution, similar clinical features were found in both groups. In CHADS_2_ group, 128 patients (35%) had a CHA_2_DS_2_-VASc score ≥ 2 (Table 1). Among them, 88 patients (69%) had age 65–75 as an additional CHA_2_DS_2_-VASc risk component to congestive heart failure, hypertension, or diabetes; 54 (42%) had female gender; 20 (16%) had age ≥ 75; and 9 (7%) had vascular disease.

**Table 1:** Clinical and echocardiographic characteristics of the study groups

Parameters	CHADS_2_ group *(n = 349)*	CHA_2_DS_2_-VASc group *(n = 221)*	*P*
Age (years)	65 ± 10	62 ± 11	0.012
Female, n (%)	93 (27)	39 (18)	0.005
Paroxysmal AF, n (%)	249 (71)	169 (76)	0.18
CHADS_2_ score	0.64 ± 0.48	0.47 ± 0.50	< 0.001
0	124 (36)	118 (53)	
1	225 (64)	103 (47)	< 0.001
CHA_2_DS_2_-VASc score	1.27 ± 0.86	0.71 ± 0.45	< 0.001
0	63 (18)	63 (29)	
1	158 (45)	158 (71)	
2	99 (28)	0 (0)	
3	28 (8)	0 (0)	
4	1 (0)	0 (0)	< 0.001
HAS-BLED score	0.44 ± 0.66	0.35 ± 0.59	0.11
Congestive heart failure, n (%)	56 (16)	28 (13)	0.26
Hypertension, n (%)	131 (38)	66 (30)	0.059
Age 65–75, n (%)	135 (39)	47 (21)	< 0.001
Age ≥ 75 years, n (%)	20 (6)	0 (0)	< 0.001
Diabetes mellitus, n (%)	15 (4)	6 (3)	0.32
Dyslipidemia, n (%)	64 (18)	41 (19)	0.95
Stroke/TIA, n (%)	0 (0)	0 (0)	–
Vascular disease, n (%)	9 (3)	0 (0)	0.003
eGFR (mL/min/1.73m^2^)	68 ± 16	69 ± 15	0.55
eGFR < 60 mL/min/1.73m^2^, n (%)	88 (25)	53 (24)	0.51
BNP (pg/mL)	93 ± 257	80 ± 201	0.53
BNP ≥200 pg/mL, n (%)	30 (9)	17 (8)	0.70
Anticoagulation, n (%)	338 (97)	215 (97)	
Warfarin, n (%)	96 (28)	67 (30)	
DOACs, n (%)	242 (69)	148 (67)	0.93
Echocardiography			
LA diameter (mm)	42 ± 7	41 ± 7	0.22
LA diameter ≥ 50 mm, n (%)	46 (13)	22 (10)	0.24
LV end-diastolic dimension (mm)	48 ± 6	48 ± 6	0.81
LV end-systolic dimension (mm)	31 ± 7	31 ± 6	0.99
LVEF (%)	62 ± 8	62 ± 7	0.64
LVEF< 50%, n (%)	18 (5)	10 (5)	0.73
Thickness of IVS (mm)	9 ± 2	9 ± 2	0.89
Thickness of LV posterior wall (mm)	9 ± 1	9 ± 1	0.85
LV mass (g)	159 ± 46	160 ± 48	0.89
LV mass index (g/m^2^)	93 ± 24	92 ± 25	0.80
LV hypertrophy, n (%)	86 (25)	47 (21)	0.35
More-than-mild MR, n (%)	17 (5)	9 (4)	0.88
LAA velocity (cm/s)	58 ± 28	61 ± 28	0.26
LAA velocity < 20 cm/s, n (%)	18 (5)	9 (4)	0.55
LASEC, n (%)	77 (22)	41 (19)	0.31
LA thrombus, n (%)	1 (0)	0 (0)	0.32

Values are mean (±SD) or number of subjects (%). *BNP* indicates B-type natriuretic peptide, *DOACs* indicates Direct oral anticoagulants, *eGFR* Estimated glomerular filtration rate, *IVS* Interventricular septum, *LAA* Left atrial appendage, *LVEF* Left ventricular ejection fraction, *MR* Mitral regurgitation, and *TIA* Transient ischemic attack

**Fig. 2:**
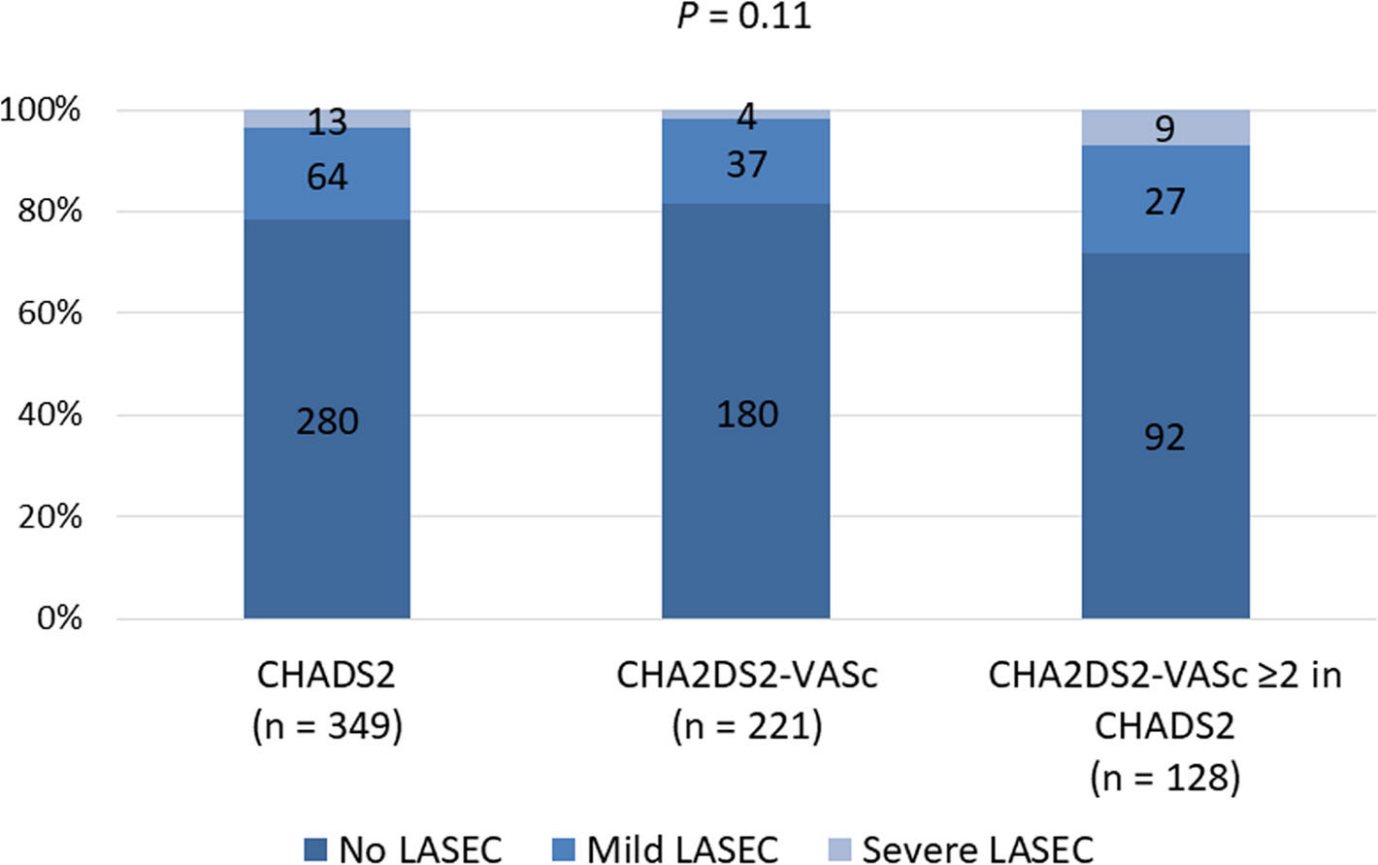
The distribution of LASEC severity in CHADS_2_ (left) and CHA_2_DS_2_-VASc (middle) groups, in addition to a group of patients with a CHADS_2_ score < 1 and with a CHA_2_DS_2_-VASc score ≥ 2 (right)

For both groups, nearly 10% of patients were shown to have significant LA enlargement and 5% to have reduced LV ejection fraction. LASEC was detected in 77 of CHADS_2_ group (22%) and in 41 of CHA_2_DS_2_-VASc group (19%). A small number of patients had LAA dysfunction (nearly 5% for both groups), and LA thrombus was found in only one patient, belonging to CHADS_2_ group. Figure 2 compares distribution of LASEC severity in CHADS_2_ and CHA_2_DS_2_-VASc groups in addition to a group of patients with CHA_2_DS_2_-VASc score ≥ 2, albeit included in CHADS_2_ group (*n* = 128). As shown, all groups included patients who had severe LASEC (4, 2, and 7%, respectively), and overall there was no statistically significant difference in LASEC severity between the groups (*P* = 0.11).

### Determinants of LASEC

Table 2 shows the results of univariate logistic regression analysis for assessing determinants of the presence of LASEC for each group. It was found that for CHADS_2_ group, parameters except female gender were significantly related to LASEC whereas for CHA_2_DS_2_-VASc group, parameters except female gender and renal dysfunction were significantly related to LASEC.

**Table 2:** Univariate logistic regression analysis for assessing determinants of LASEC

	CHADS_2_ group			CHA_2_DS_2_-VASc group		
Parameters	Odds ratio	95% CI	*P*	Odds ratio	95% CI	*P*
Female	1.56	0.91–2.70	0.11	1.99	0.89–4.43	0.092
Non-paroxysmal AF	7.00	4.04–12.2	< 0.001	4.40	2.14–9.07	< 0.001
eGFR < 60 mL/min/1.73m^2^	2.18	1.27–3.76	0.005	1.63	0.77–3.43	0.20
BNP ≥200 pg/mL	6.61	3.02–14.5	< 0.001	4.61	1.66–12.8	0.003
LA diameter ≥ 50 mm	3.71	1.94–7.09	< 0.001	3.61	1.42–9.16	0.007
LVEF < 50%	8.18	2.96–22.6	< 0.001	7.54	2.02–28.1	0.003
LV hypertrophy	3.35	1.95–5.75	< 0.001	3.08	1.47–6.43	0.003

All abbreviations are as in Table 1

Multivariate logistic regression analysis (Table 3), adjusted for parameters that were of statistical significance in the univariate analysis (*P* < 0.05), demonstrated that for CHADS_2_ group, non-paroxysmal AF, BNP elevation, and LV hypertrophy were significant independent determinants of LASEC, and that for CHA_2_DS_2_-VASc group, non-paroxysmal AF and LV hypertrophy were significant independent determinants of LASEC.

**Table 3:** Multivariate logistic regression analysis for assessing determinants of LASEC

	CHADS_2_ group			CHA_2_DS_2_-VASc group		
Parameters	Odds ratio	95% CI	*P*	Odds ratio	95% CI	*P*
Female	–	–	–	–	–	–
Non-paroxysmal AF	5.65	3.08–10.5	< 0.001	3.38	1.51–7.54	0.003
eGFR < 60 mL/min/1.73m^2^	1.76	0.93–3.34	0.082	–	–	–
BNP ≥200 pg/mL	3.42	1.29–9.06	0.013	1.76	0.49–6.33	0.39
LA diameter ≥ 50 mm	1.38	0.63–3.03	0.42	1.42	0.48–4.20	0.53
LVEF < 50%	2.53	0.71–9.09	0.15	3.10	0.63–15.4	0.17
LV hypertrophy	2.26	1.19–4.28	0.013	2.53	1.13–5.70	0.025

All abbreviations are as in Table 1

**Fig. 3:**
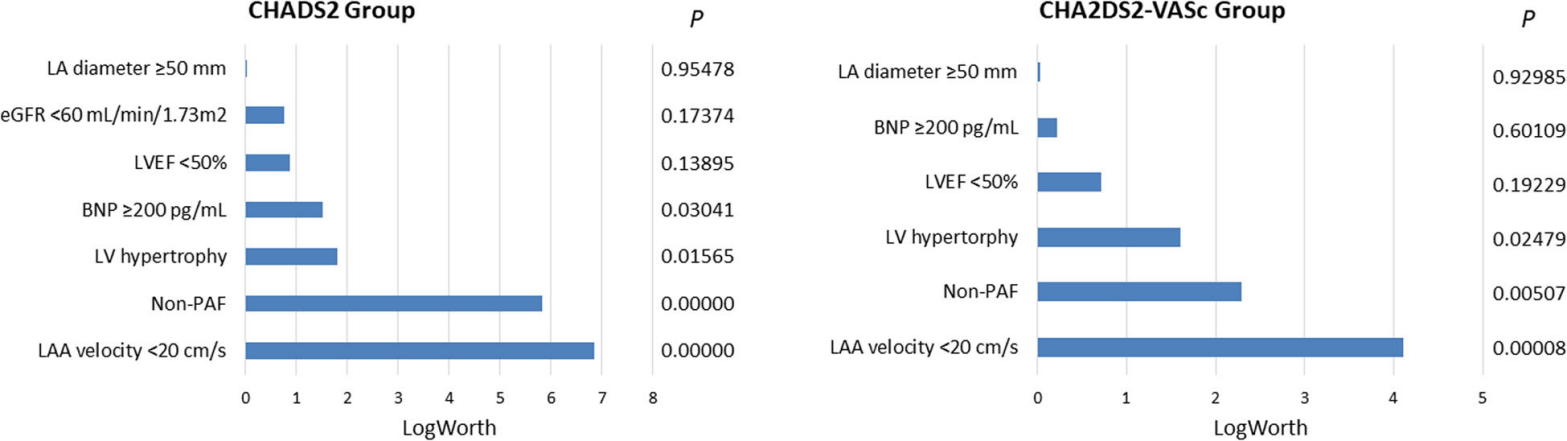
A LogWorth of 2 is identical to “P = 0.01”. All abbreviations are as in Table 1

Figure 3 compares contribution of clinical and echocardiographic parameters to LASEC detection, which is based on the multivariate analysis as in Table 3, with an additional covariate of “LAA velocity <20 cm/s” being included into the model. It was found that for both CHADS_2_ and CHA_2_DS_2_-VASc groups, LAA velocity < 20 cm/s and non-paroxysmal AF were exceeding a LogWorth value of 2, which is identical to *P* < 0.01.

## Discussion

It was demonstrated in our population that a considerable proportion of patients with low TE risk scores had LASEC, that clinical and echocardiographic parameters did not differ as much between CHADS_2_ and CHA_2_DS_2_-VASc groups, and that on the multivariate analysis, LASEC occurrence was related to non-paroxysmal AF, BNP elevation (BNP ≥200 pg/mL), or LV hypertrophy.

### Previous studies on LASEC and thromboembolic risk scores

There are several reports on the relationship between TEE findings and TE risk scores. In most cases, the prevalence of LASEC and/or LAA dysfunction was shown to increase accordingly with increases in CHADS_2_ and CHA_2_DS_2_-VASc scores [6–8]. One explanation for this association is that elevation of TE risk scores is more likely to be associated with cardiac conditions predisposed to thrombus formation such as LA enlargement and LV systolic dysfunction [14–16]. In a different view point, increases in CHADS_2_ and CHA_2_DS_2_-VASc scores may enhance production of various inflammatory cytokines exerting as prothrombotic substrates [26, 27].

### LASEC occurrence under low TE risk scores

There are several studies on LASEC occurring under low TE risk scores [6, 9, 10, 15]. We observed that approximately 20% of the low TE risk score patients had LASEC, the number of which was similar to that reported previously [6, 15]. Although our data suggest that AF persistence and LV hypertrophy are associated with LASEC production, the pathological basis for SEC is quite complex, with various factors being interplay [28]; in fact, some investigators failed to find relationship between TE risk scores, LASEC, and LAA velocity [22].

Yao et al. reported that an elevated plasma homocysteine could be a risk of LA thrombus in nonvalvular AF patients [9]. Homocysteine seems to accelerate arterial and venous thrombosis through biological damage to vascular endothelium by generating oxidative stress, reducing NO-production, and inducing inflammatory response [9]. Kimura et al. used computed tomography for 3-dimesinal construction of the atrium to assess relationship between LAA morphologies (cactus, cauliflower, chicken-wing, and windsock) and a risk of stroke. They found that the cauliflower type was mostly related to the prior stroke especially in those with low CHADS_2_ scores [29].

LASEC occurring in our population appears to result from the difference in individual TE risk components (hypertension, diabetes, etc.), rather than the difference in the scores themselves. This might be substantiated by the finding in Table 4 that congestive heart failure was more common in patients with LASEC than those without, whereas other TE risk components such as hypertension did not show such differences. Congestive heart failure is a syndrome that is usually associated with cardiac changes leading to the development of LASEC [11–15].

**Table 4:** Prevalence of thromboembolic risk factors in patients with LASEC and those without

	CHADS_2_ group			CHA_2_DS_2_-VASc group		
Parameters	LASEC absent *(n = 272)*	LASEC present *(n = 77)*	*P*	LASEC absent *(n = 180)*	LASEC present *(n = 41)*	*P*
Congestive heart failure, n (%)	26 (10)	30 (39)	< 0.001	13 (7)	15 (37)	< 0.001
Hypertension, n (%)	104 (38)	27 (35)	0.61	57 (32)	9 (22)	0.21
Age 65–75	104 (37)	32 (40)	0.64	41 (22)	6 (14)	0.21
Age ≥ 75 years, n (%)	17 (6)	3 (4)	0.41	0 (0)	0 (0)	–
Diabetes mellitus, n (%)	14 (5)	1 (1)	0.10	6 (3)	0 (0)	0.11
Stroke/TIA, n (%)	0 (0)	0 (0)	–	0 (0)	0 (0)	–
Vascular disease, n (%)	5 (2)	4 (5)	0.13	0 (0)	0 (0)	–
Female, n (%)	67 (24)	26 (34)	0.12	28 (16)	11 (27)	0.10

All abbreviations are as in Table 1

The finding of LV hypertrophy being stratified as a predictor of LASEC better than other parameters such as LA diameter and LV ejection fraction was surprising. This may be relate, for one thing, to the fact that LV hypertrophy is often associated with LA enlargement [30], potentially leading to the occurrence of LASEC; in fact, for CHA2DS2-VASc group, 10 patients (21%) with LV hypertrophy had LA enlargement, and 9 (41%) with LA enlargement had LASEC. In addition, the relatively limited number of patients who had either LA diameter ≥50 mm or LV ejection fraction <50% (Table 1) might exclusively contributed to LV hypertrophy that emerged as a significant correlate of LASEC.

### Clinical implications

One message in the present study is to determine what parameters, except TEE ones, would be responsible for LASEC that occurs in patients with low TE risk scores. The exception of TEE parameters was based on the fact that TEE is a semi-invasive procedure with its application as a screening tool being limited. As shown in Fig. 3, “LAA velocity <20 cm/s” and “non-paroxysmal AF” are comparable in contributing to LASEC detection in both groups. This suggests that AF chronicity, even without support from TEE, becomes the best  marker of LASEC occurrence, and particularly, this finding would be supporting the recommendation by Puwanant et al. that a screening TEE should be performed in patients with a CHADS_2_ score of 0 whose AF is persistent [6].

### Limitations

The present study is subject to the limitations inherent to a single center study. All clinical and echocardiographic data were obtained retrospectively and thus a certain kind of misclassification might be inevitable. Another limitation was that the duration of AF and the adequacy of anticoagulation could not be reliably extracted from the patient records, which might result in overestimation of the number of non-paroxysmal AF patients. However, LASEC represents not only a history of AF but also condition of the atrial tissue [31, 32], and most of our patients were on anticoagulation that was religiously monitored for hemorrhagic status with reference to the INR. Finally, this study consists of a small number of patients and thus our findings may not be generalized to other population.

## Conclusions

We investigated clinical and echocardiographic parameters that would determine LASEC formation on nonvalvular AF patients with low CHADS_2_ or CHA_2_DS_2_-VASc scores. About 20% of the patients were found to be associated with LASEC. With results of the multivariate analysis taken into account, information on AF chronicity, BNP, and LV hypertrophy might help identify patients at risk for thromboembolism, although large-scale studies are necessary to confirm our observations.

## Data Availability

All data generated or analyzed during this study are included in this published article [and its supplementary information files].

## Abbreviations

AF: Atrial fibrillation
BNP: B-type natriuretic peptide
eGFR: Estimated glomerular filtration rate
INR: International normalized ratio
LA: Left atrial
LAA: Left atrial appendage
LASEC: Left atrial spontaneous echo contrast
LV: Left ventricular
TE: Thromboembolic
TEE: Transesophageal echocardiography
